# Endotoxin-activated microglia injure brain derived endothelial cells via NF-κB, JAK-STAT and JNK stress kinase pathways

**DOI:** 10.1186/1476-9255-8-7

**Published:** 2011-03-07

**Authors:** Rachid Kacimi, Rona G Giffard, Midori A Yenari

**Affiliations:** 1 Dept. Neurology, University of California, San Francisco & San Francisco Veterans Affairs Medical Center, San Francisco 94121 USA; 2 Dept. Anesthesia Department, Stanford University Medical Center, Stanford 94305 USA

## Abstract

**Background:**

We previously showed that microglia damage blood brain barrier (BBB) components following ischemic brain insults, but the underlying mechanism(s) is/are not well known. Recent work has established the contribution of toll-like receptor 4 (TLR4) activation to several brain pathologies including ischemia, neurodegeneration and sepsis. The present study established the requirement of microglia for lipopolysaccharide (LPS) mediated endothelial cell death, and explored pathways involved in this toxicity. LPS is a classic TLR4 agonist, and is used here to model aspects of brain conditions where TLR4 stimulation occurs.

**Methods/Results:**

In monocultures, LPS induced death in microglia, but not brain derived endothelial cells (EC). However, LPS increased EC death when cocultured with microglia. LPS led to nitric oxide (NO) and inducible NO synthase (iNOS) induction in microglia, but not in EC. Inhibiting microglial activation by blocking iNOS and other generators of NO or blocking reactive oxygen species (ROS) also prevented injury in these cocultures. To assess the signaling pathway(s) involved, inhibitors of several downstream TLR-4 activated pathways were studied. Inhibitors of NF-κB, JAK-STAT and JNK/SAPK decreased microglial activation and prevented cell death, although the effect of blocking JNK/SAPK was rather modest. Inhibitors of PI3K, ERK, and p38 MAPK had no effect.

**Conclusions:**

We show that *LPS-activated microglia promote BBB disruption *through injury to endothelial cells, and the specific blockade of JAK-STAT, NF-κB may prove to be especially useful anti-inflammatory strategies to confer cerebrovascular protection.

## Background

Microglia are the brain's resident immune cell, and are among the first to respond to brain injury. Microglia are rapidly activated and migrate to the affected sites of neuronal damage where they secrete both cytoxic and cytotrophic immune mediators [1]. Homeostasis of the brain's microenvironment is maintained by the blood-brain barrier (BBB), formed by endothelial cell tight junctions. The BBB is now recognized to comprise complex and dynamic cellular systems, whereby astrocytes, microglia, perivascular macrophages, pericytes and the basal membrane interact with endothelial cells tight junctions, and serve as a controlled functional gate to the brain [2]. Endothelial cell permeability, activation and injury play a critical role in the progression of disease processes including inflammation, atherosclerosis, and tumor angiogenesis [3]. Microglia are assumed to play a crucial role in the formation and homeostasis of the BBB [4]. In response to potential pathogen invasion, microglia react to destroy infectious agents before they damage the neural tissue. Moreover, microglial activation is crucial in the progression of multiple inflammatory diseases via the release of inflammatory mediators such as cytokines, NO, and prostaglandins [1,5].

We previously showed that microglia potentiated injury to BBB components following ischemia like insults, and pharmacological inhibition of microglia reduced BBB disruption in an experimental model of stroke [6]. Here we expand on these findings to identify underlying mechanisms of this microglial toxicity. Since many insults are capable of damaging endothelial cells in the absence of microglia, we focused on a model of endothelial cell death that occurred only in the presence microglia to better understand their role in potentiating injury.

## Methods

### Chemicals and reagents

All reagents were high grade and were purchased from Sigma with the following exceptions. RPMI, DMEM, Calcein and ethidium homodimer and other culture reagents were purchased from Invitrogen Inc (Grand Island, NY, USA) and the UCSF cell culture facility (UCSF, San Francisco, CA). Fetal bovine Serum Defined (FBS) was purchased from Hyclone Laboratories (Logan, UT, USA). PD98059, a MEK inhibitor; SP600 125, a JNK inhibitor; wortmanin an inhibitor of PI3 kinase and pyrrolidinecarbodithoic acid (PDTC), a NF-κB inhibitor); AG490, a JAK2-STAT inhibitor were purchased from Calbiochem (San Diego, CA). LPS (*Escherichia coli*, O26:B6), aminoguandine, apocynin, allopurinol, minocycline, N(omega)-hydroxy-L-arginine (NOHA), indomethacin and amino-3-morpholinyl-1,2,3-oxadiazolium chloride (SIN-1) were purchased from Sigma (St Louis, MO). Drugs were dissolved in DMSO or ethanol and stored at -20°C and either used (final concentration of vehicle 0.1% (v/v or dried down and resuspended in PBS/0.1% bovine serum albumin (BSA). Mitogen activated kinase (MAPK) Anti-phospho-ERK monoclonal antibody (mAb), anti-ERK polyclonal antibody (#4370), anti-phospho-p38 MAPK mAb (# 4631), anti-phospho-JNK/SAPK mAb (#4668) were from Cell Signaling Technology (Danvers, MA); anti-NF-κBp65 (# SC-8008), anti-IκBα (# SC-1643) and respective horseradish peroxidase-coupled secondary antibodies were purchased from Santa Cruz (Santa Cruz, CA) and. Antibodies against iNOS ( # 61043), iNOS positive control lysates (#611473) were from BD Biosciences (BD Biosciences, Lexington, KY).

## Cell culture

### BV2 cells

The immortalized mouse microglia cell line, BV2, originally generated by Blasi and colleagues [7], were obtained from Dr. Theo Palmer. These cells were exhaustively shown to exhibit many phenotypic and functional properties of reactive microglia cells and are suitable model of inflammation [8]. Cells were grown and maintained in RPMI supplemented with 10% fetal bovine serum and antibiotics (penicillin/streptomycin, 100U/ml). Under a humidified 5% CO_2_/95% air atmosphere and at 37°C, cells were plated in 75 cm^2 ^cell culture flask (Corning, Acton, MA, USA) and were split twice a week. For the experiments, cells were plated on 6-well dishes (1-2 × 10^6^cells/well).

### bEND.3 cells

The immortalized mouse brain microvascular endothelial cell line, bEND.3, was purchased from American Type Culture Collection (Manassas, VA, USA). These cells were derived from mouse brain endothelial cells prepared from cerebral capillaries of C57BL/6 mice [9]. Cells were grown in Dulbecco's modified Eagle's medium (DMEM) supplemented with 450 mg/dl glucose, 10% fetal bovine defined, and antibiotics.

Cocultures of BV2 and bEND.3 cells were generated by growing bEND.3 cells to confluence in DMEM with serum. BV2 cells were then seeded on the top of the monolayer with the bEND.3 cells and allowed to adhere for 24 hours before each experimental design. *A ratio of 1:10 (BV2: bEND.3 cells) was used to model the relative proportions observed in vivo*.

Each cell type described above were characterized by morphological appearance, viability with trypan blue or calcein, immunocytochemical staining or Western blotting using antibodies that recognizes specific markers (VW Factor, PECAM-1 and claudin-5 for bEND.3; IBA lectin for BV2 cells as previously described [6,10,11].

### Experimental protocols

#### Cell treatment

Cells were cultured to approximately 80% confluence, and fresh serum-free media was added for 4-24 h before LPS or inhibitors treatments. All inhibitors were applied 1 h before experimental treatment. Of note, we did preliminary *dose finding *and toxicity studies for all the selective inhibitors used. We selected optimal concentrations that both inhibited NO generation without cytotoxic effect on cells as indicated for each drug accordingly.

#### Fluorescence microscopy

Fluorescence immunocytochemistry was performed on cells as previously described [12]. After washing, cells were fixed with acetone/methanol (1:1) 5 min at -20°C. Alternatively, cells were fixed in 4% paraformaldehyde for 30 min at room temperature. The cells were then washed twice with PBS containing 0.2% Triton X-100 for 15 min. Nonspecific binding sites were blocked in blocking buffer (2% BSA and 0.2% Triton X-100 in PBS) for 2 hr. The cells were incubated with primary antibody specific marker for the vascular unit cells as indicated at 1:100 dilution in blocking buffer overnight at 4°C and then washed three times with blocking buffer, 10 min per wash. The cells were incubated with FITC- or Texas Red-conjugated secondary antibodies (Jackson ImmunoResearch, West Grove, PA) at 1:100 dilution in blocking buffer at RT for 1 h, then washed 2 times in blocking buffer, and one time in PBS, 10 min per wash. Fluorescence was visualized with an epifluorescence microscope (Zeiss Axiovert; Carl Zeiss Inc), and images were obtained on a PC computer using Axiomatic software (Zeiss Inc).

#### NO measurement

LPS or vehicle was then added as described above, and cells were returned to the incubator. After incubation for 24 h, aliquots of the incubation media were removed and either stored at -80°C or used immediately for nitrite content analysis. Accumulation of NO in cultures media was determined by the Greiss reagent using nitrite as standard as previously described [13-15].

#### Immunoblotting

After each treatment period, cells plated on 6 well or 60-mm dishes were washed with cold phosphate buffered saline, and scraped into 500 μl lysis buffer consisting of 20 mM Tris, pH7.5, 150 mM NaCl, 1% Triton X-100, 0.5% NP-40, 1 mM EDTA, 1 mM EGTA, 1 mM sodium orthovanadate, 1 mM phenylmethylsulfonylfluoride (PMSF), 50 mM NaF, and 5 mg/ml aprotinin. Lysates were sonicated and centrifuged at 10,000 × *g *for 5 min. The supernatant was collected and either used immediately or frozen at -80°C. Protein concentration was determined using the BCA protein assay (Pierce, Rockford, IL), and equal amounts of protein were loaded per lane onto 10-12% sodium dodecylsulfate-polyacrylamide gels, and were electrophoresed (SDS-PAGE) as previously described [12,16]. Gels were then transferred onto enhanced chemiluminescence (ECL)-nylon membranes in transfer buffer containing 48 mM Tris, 150 mM glycine, and 10% methanol using a Transblot apparatus (Biorad, Hercules, CA, USA) at 100 V for 1 hr at 4°C. The membranes were saturated in 10 mM Tris, pH7.4, 150 mM NaCl, and 0.1% Tween-20, and 5% non-fat dry milk for 1 hr at room temperature and then probed with specific polyclonal antisera for iNOS the same buffer for 1 h at room temperature with gentle agitation. anti-phospho-p38 MAPK mAb, anti-phospho-JNK mAb, *and anti-phospho JAK2 *rabbit polyclonal antibodies were from Cell Signaling Technology (Danvers, MA). For all antibodies used working dilution was (1:500 and 1;1000) for rabbit and mouse primary antibodies respectively. Membranes were washed three times with 10 mM Tris, 150 mM NaCl, and 0.1% Tween-20. Bound antibodies were identified after incubation with peroxidase-conjugated anti-rabbit antibodies (1:2000 dilution in saturation buffer) for 1 h at room temperature. Membranes were then rewashed three times and the position of the individual proteins was detected by chemiluminescence ECL according to the manufacturer's instruction

#### Assessment of IκB-α degradation and NF-κB nuclear translocation

Cytoplasmic and nuclear extracts were prepared as previously described [17]. IκBα in cytoplasmic extracts and NF-κB subunit p65 in nuclear extracts were detected by Western blot using specific antibodies anti-NF-κBp65 and anti-IκBα [18]. *We also assessed NF-κB activation using anti- phospho NF-κB p65 subunit antibody (rabbit polyclonal, Cell Signaling Technology) by western blot*.

#### Cell viability assays

MTT was used to assay cell viability. Trypan blue exclusion and calcein/ethidium homodimer dual stain were also used to morphologically assay for cell viability (Live/dead, calcein/ethidium homodimer dual stain) as previously described [12,14]. *Estimates of relative bEND.3 and BV2 cell viability were made from manual counts from cultures labelled with calcein and appropriate cell type markers, and manual counts were made from 5 non-overlapping fields*.

#### Statistical analysis

Data are presented as mean ± SEM. Significant differences were determined by either Student's two-tailed *t*-test for comparison of the means of two samples or analysis of variance (ANOVA) for the comparison of more than two sample means followed by Newman-Keuls post-hoc testing for multiple comparisons among sample means. The significance level was set at *P*< 0.05.

## Results

### LPS dose response and NO generation

**Figure 1:**
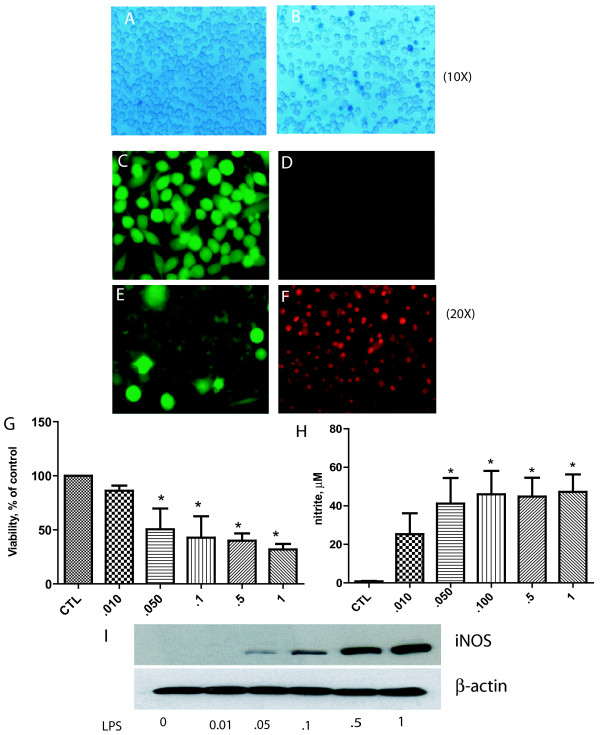
**LPS induces BV2 cell death**. Compared to control BV2 cells (A, C, E), LPS exposure for 24 hours led to increased cell death in a dose dependent manner (B, D, F, G). Fewer numbers of BV2 cells (B) are observed after LPS (*1 μg/mL*) treatment compared to those given vehicle (A) (trypan blue stain). Calcein stained cells reveal viable cells in green (C, D). Double staining with calcein (live cells in green) and ethidium homodimer (nuclear stain of dead cells in red) (E, F). **LPS induces generation of iNOS and NO in a dose dependent manner in microglia**. BV2 cells were incubated in vehicle (CTL) or LPS for 24 h. Thereafter, cells were harvested, and lysates were used for Western blot. Nitrite levels, a measure of NO generation, was determined from supernatants. LPS reduced BV2 cell viability (G, n = *independent observations*) and increased NO generation (H, n = 12 *independent observations*) in a dose dependent manner. iNOS protein was similarly increased with LPS concentration (I). Shown is a representative blot. *P < 0.05 versus control.

We investigated the effects of a proinflammatory stimulus on BV2 cells. Our first observation showed that LPS induced injury to BV2 cells as detected by analysis of cell morphology and viability assays (Figure 1A-G). We also found that LPS (0.01-1 μg/ml) induced NO production (Figure 1H), which was dose dependent and inversely related to cell viability. LPS also induced iNOS protein in a dose dependent manner (Figure 1I). LPS also increased the levels of ROS generation and other proinflammatory markers COX-2 and TNFα (not shown). Thus, all subsequent experiments used a LPS concentration of 1 μg/ml.

### LPS does not affect endothelial cell viability or NO/iNOS induction

**Figure 2:**
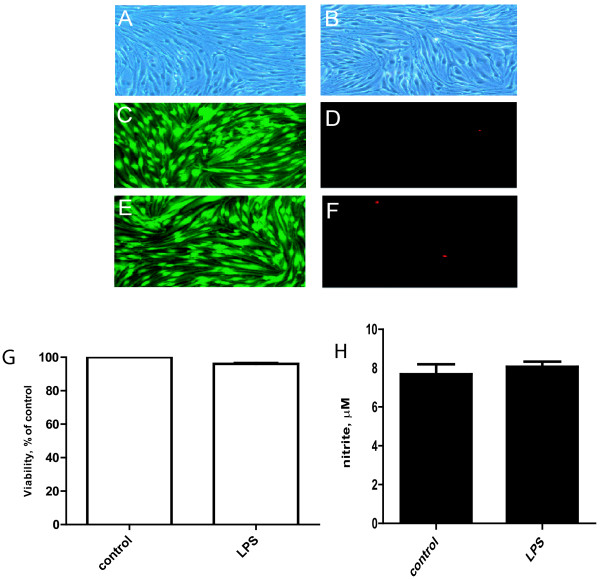
**LPS does not affect iNOS expression and cell survival in endothelial cells**. bEND.3 cells exposed to 1 μg/ml LPS for 24 h fail to experience any cell death (A, C, *D*-control, B, *E*, F-LPS treatment). Shown are trypan blue (A, B), calcein (C, E) and ethidium homodimer (D, F) stains. LPS has no effect on bEND.3 cell viability as assessed by MTT staining (H) and NO generation (G). LPS also fails to induce iNOS protein in bEND.3 cells (I). iNOS protein is readily induced by LPS in BV2 cells as a positive control. Data are representative of 3-5 experiments.

In contrast, LPS (1 μg/ml) had no direct effect on bEND.3 cell viability, and did not increase NO or induce iNOS (Figure 2). The baseline levels of NO present in the media of bEND.3 cells were likely generated by eNOS, which is known to be constitutively expressed in these cells.

### NO donors affect BV2 cells in a manner similar to LPS

**Figure 3:**
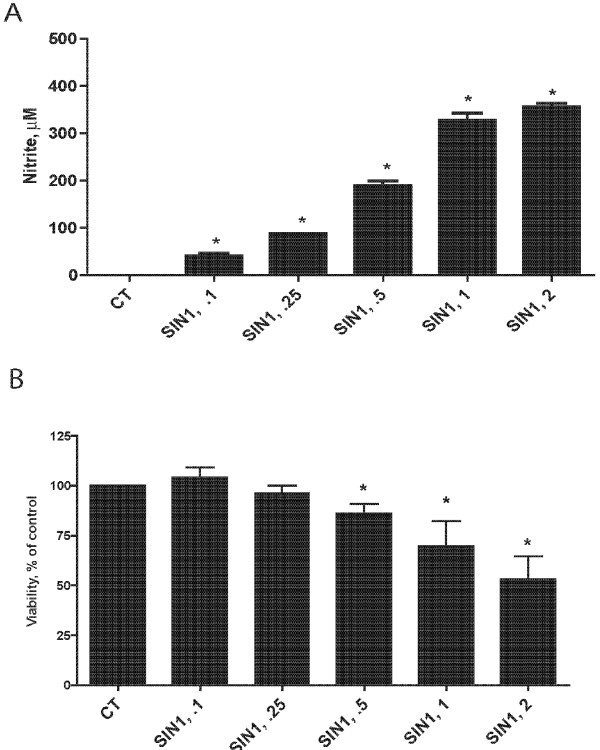
**SIN-1, a NO donor shows similar patterns on viability and NO accumulation in microglia as LPS treatment**. BV2 cells were incubated in increasing doses of SIN-1 for 24 hr. NO accumulation as determined by the Greiss reagent (A) increased in a dose dependent manner. Viability, as assessed by light microscopy and MTT quantification, also decreased, but only at concentrations of 0.5 mM or greater. n = 6 *independent observations*, *P < 0.05 versus control

Because LPS stimulated NO generation in BV2 cells, we explored whether a NO donor behaved in a similar fashion. Accordingly, BV2 cells were treated with serial doses of the NO donor SIN-1 for 24 h. Like LPS, SIN-1 *(0.1-1 mM) *dose dependently increased NO generation and reduced BV2 cell viability (Figure 3). While SIN-1 did not alter cell viability at the lowest doses studied, NO accumulation was more dramatically affected.

### Differential effect of BV2 viability & NO/iNOS generation by various immune inhibitors

**Figure 4:**
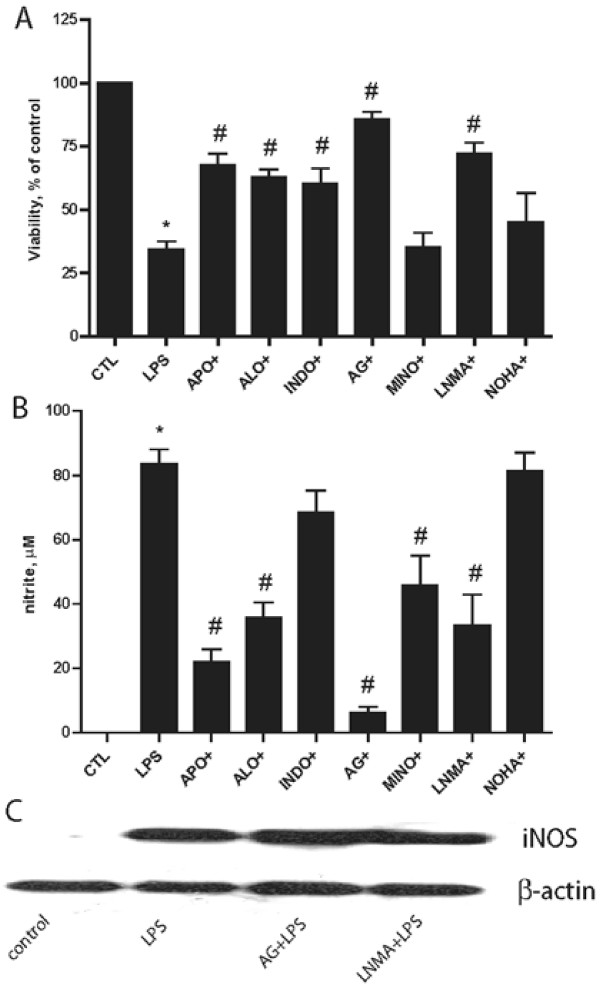
**NOS and ROS inhibitors improve microglial viability and reduce NO accumulation**. A panel of NO (AG, *1 mM*; LNMA, *1 mM*) and ROS (APO, *1 mM*; ALO, *50 μM*; INDO, *10 μM*) inhibitors as well as minocycline (MINO, *10 μM*) known to have anti-inflammatory properties and NOHA (an arginase inhibitor, *10 μM*) were studied in BV2 cells exposed to LPS (1 μg/ml). BV2 cell viability as assessed by MTT showed that all of the ROS and NOS inhibitors protected the cells, but not MINO or NOHA (A). LPS-induced NO in BV2 cells was attenuated by some (APO, ALO, AG, MINO, LNMA) but not all inhibitors (B). Neither NOS inhibitor inhibited LPS induced increases in iNOS (C), shown is a representative blot of at least 3 independent experiments. (AG: aminoguanidine, a relatively selective iNOS inhibitor; LNMA: L-NNMA, a non selective NOS inhibitor APO: apocynin, a NADPH oxidase inhibitor; ALO: allopurinol, a xanthine oxidase inhibitor; INDO: indomethacin, a COX inhibitor) n = 12 *independent observations*, *P < 0.0001 versus control; #P < 0.0001 versus LPS.

In order to determine whether the increase in NO by LPS is specific to iNOS; we tested the effect of various immune inhibitors on BV2 cell viability and NO accumulation. We found that NOS (aminoguanidine and L-NMMA) and ROS (apocynin, allopurinol, indomethicin) inhibitors all reduced LPS-induced cell death in BV2 cells (Figure 4A). Interestingly, aminoguanidine (AG, a relatively selective iNOS inhibitor, *1 mM*) and L-NMMA (a non selective NOS inhibitor, *100 μM*) both abrogated NO accumulation, as did apocynin (APO, a NADPH oxidase inhibitor, *1 mM*), allopurinol (ALLO, a xanthine oxidase inhibitor, *50 μM*) and minocycline (MINO, *10 μM*) an antibiotic known to have multiple anti-inflammatory properties [19], but not COX-2 (indomethacin, *10 μM*) or arginase (NOHA, *10 μM*) inhibitors (Figure 4B). Neither NOS inhibitor had an effect on iNOS induction elicited by LPS (Figure 4C), consistent with these compounds' ability to inhibit NOS activity but not protein levels.

### NF-κB, JAK/STAT and JNK are involved in LPS activation of BV2 cells

**Figure 5:**
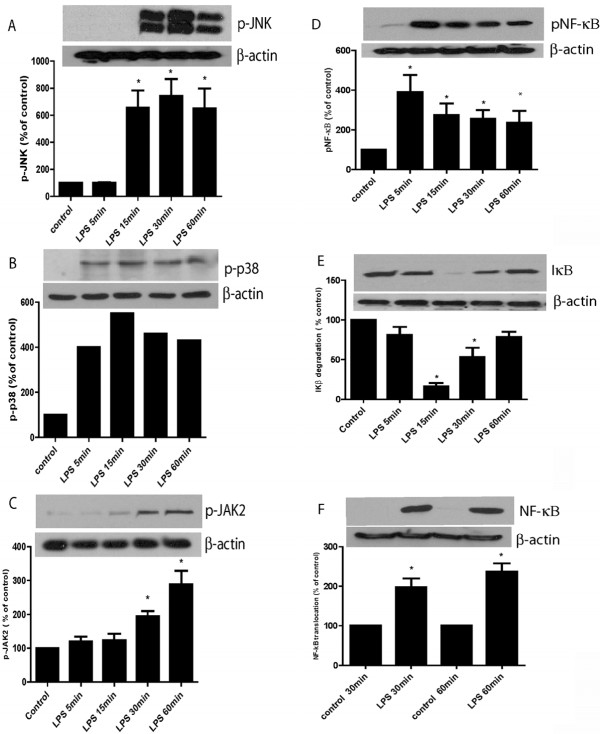
**LPS activates JNK, p38 MAPK, JAK-STAT and NF-κB in microglia**. BV2 cells were treated with LPS, and cell lysates were collected for Western blot analysis at the various times shown. LPS activated JNK (A, shown using a phospho specific antibody against phosphorylated JNK, p-JNK), the p38 MAPK ( p-p38) (B) and JAK-STAT, as evidenced by phosphorylated JAK2 ( p-JAK2) (C). NF-κB was also activated as shown by increased phosphorylation of its p65 subunit (D), decreasing levels of its inhibitor protein IκB (E) and increased nuclear accumulation of its p65 subunit (F). Shown are representative blots, plus bar graphs for quantitative comparison using densitometry (A -F). Data are mean ± SEM, n = 3-5 independent experiments. *P < 0.05. Optical densitometric values were normalized to β-actin as a housekeeping control, and are expressed as percentage of controls.

Transcription factors NF-kappa B (NF-κB) and mitogen-activated protein kinase (MAPK) are known to play upstream roles in NO/iNOS signaling. To determine which of these pathways is activated by LPS, BV2 cells were treated with LPS and respective inhibitors, then collected at different timepoints ranging from 5-60 min. Western blot analysis using phospho specific antibodies showed that LPS triggered an early (5 min) increase in the activation of stress activated p38 MAPKs, whereas c-Jun N-terminal kinases (JNKs/SAPKs) *and JAK-STAT *activation was detected at 30 min (Figure 5). LPS also induced degradation of I-κB with increases in nuclear NF-κB expression by 30 min *and phosphorylated NF-kB was observed as early as 5 min*.

**Figure 6:**
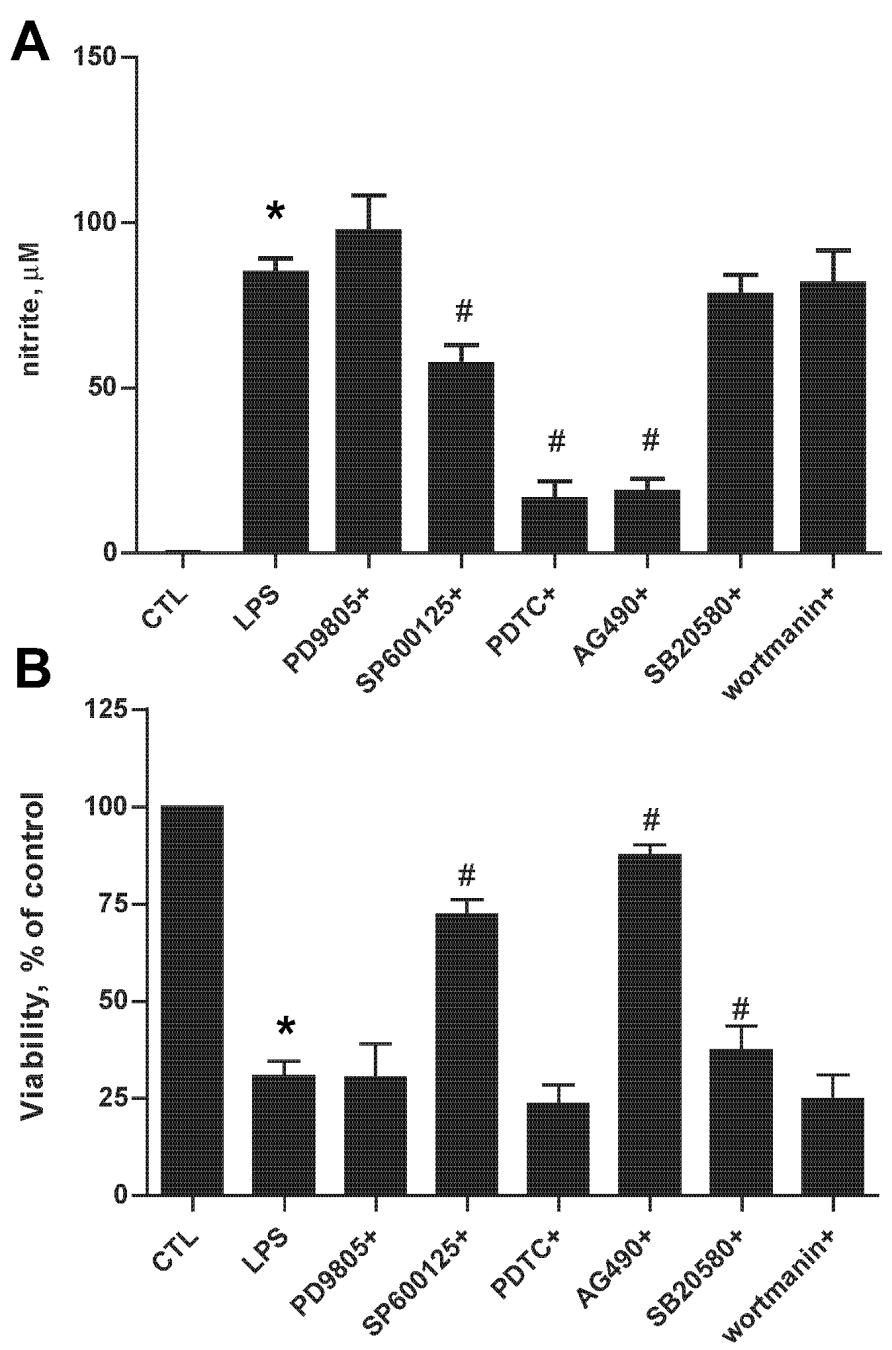
**A: NF-κB, JNK and JAK-STAT inhibition prevent LPS- induced iNOS in BV2 cultured alone**. BV2 cells were stimulated with LPS in the presence of inhibitors against MEK1 (PD9805, *20 μM*), JNK(SP600125, *10 μM*), NF-κB (PDTC, *50 μM*), JAK-STAT (AG490, *10 μM*), p38 MAPK (SB20580, *10 μM*) or PI3K (wortmannin, *100 nM*). *Inhibition of JNK, NF-κB and JAK-STAT reduced NO accumulation, whereas inhibition of MEK1, p38 MAPK and PI3K did not (A). Inhibition of JNK, JAK-STAT and p38 MAPK all protected against LPS-induced toxicity, but inhibition of MEK1, NF-κB or PI3K did not (B)*. (n = 12 *independent observations*), *P < 0.05 versus control, #P < 0.05 versus LPS.

To further assess the functional significance of these pathways in iNOS induction and NO accumulation by LPS, we studied a panel of inhibitors. Pyrodinyl dithiocarbamate (PDTC, *50 μM*) to inhibit NF-κB and AG490 (*10 μM*), a JAK-STAT inhibitor both abrogated NO accumulation, while the PI3K inhibitor wortmanin (*100 nM*), the MEK1 inhibitor PD98050 (*20 μM*) and the p38 MAPK inhibitor SB203580 (*10 μM*) did not. However, the JNK kinase inhibitor SP600125 (*10 μM*) only partially prevented NO accumulation (Figure 6B). On the other hand, while PI3K, MEK1 and p38 MAPK inhibition did not prevent cell death, JAK/STAT, and JNK kinase pathway inhibition protected BV2 cells from LPS-induced injury (Figure 6A).

### LPS induces endothelial cell death in the presence of microglia. Reversal by NOS and ROS inhibition

**Figure 7:**
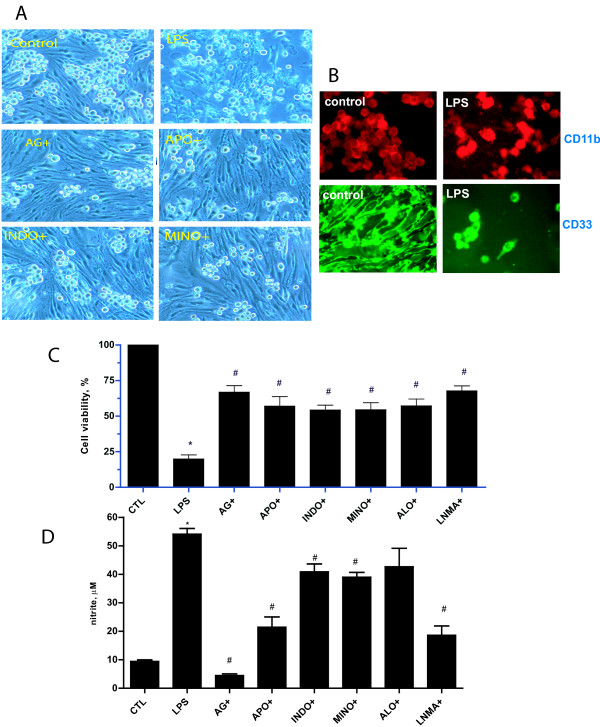
**Microglia increase endothelial cell death due to LPS, reversal by NOS and ROS inhibitors**. While LPS did not affect bEND.3 cells alone, when cultured with BV2 cells, LPS increased cell death and monolayer disruption of primarily bEND.3 cells (Panel A, LPS) compared to control cocultures (Panel A, Control). The majority cell type that succumbed to LPS was the bEND.3 rather than BV2 cells. Treatment with aminoguanidine (Panel A, AG+), apocynin (Panel A, APO+), indomethacin (Panel A, INDO+) or minocycline (Panel A, MINO+) all prevented this monolayer disruption. To determine which cell type succumbed to LPS exposure, cocultures of bEND.3 and BV2 cells were prepared and exposed to LPS for 24 h. Immunostains of cell type markers showed that endothelial cells (Panel B, CD33, green) were primarily affected compared to BV2 cells (CD11b, red), as more BV2 cells remained post LPS treatment than bEND.3 cells. Panels C & D summarize the effect of various NOS (AG, LNMA), ROS (APO, INDO, ALO) and inflammatory (MINO) inhibitors on LPS-induced cell viability (B) and NO accumulation (C). CTL: control cultures treated with vehicle, AG: aminoguanidine (1 mM), LNMA: L-NMA (1 mM); APO: apocynin (1 mM), ALO: allopurinol (1 mM); INDO: indomethacin (50 μM). (n = 4-6 *independent observations*, *P < 0.05 vs. control, #P < 0.05 versus LPS.

While LPS was not directly toxic to bEND.3 cells, cocultures of bEND.3 cells with BV2 cells led to LPS induced injury to bEND.3 cells (Figure 7A-C) and NO accumulation (Figure 7D). This toxic effect seemed to require cell-cell interactions, since conditioned media from LPS activated BV2 cells failed to induce bEND.3 cell injury (data not shown). The proportion of cell death in these cocultures was mostly the bEND.3 cells, as bEND.3 monolayer integrity was almost completely disrupted by LPS, but BV2 cells seemed relatively spared (Figure 7A). The proportion of remaining BV2 cells was about 20-30%, but overall cell death was 70-80% (Figure 7 B-C). Thus, LPS stimulation led to death of mostly bEND.3 cells. Pretreatment with NOS (L-NMMA and aminoguanidine) and ROS inhibitors (apocynin and allopurinol) markedly prevented cell death and b.END3 monolayer disruption in this experimental model. Similarly, anti-inflammatory drugs minocycline and inodmethacin protected from LPS induced injury and attenuated NO generation. These data implicate the cytotoxicity imposed by LPS activated microglia, and that this toxicity is likely mediated by reactive nitrogen and oxygen species.

### LPS activated microglia induce endothelial cell death via NF-κB, JAK-STAT and JNK

**Figure 8:**
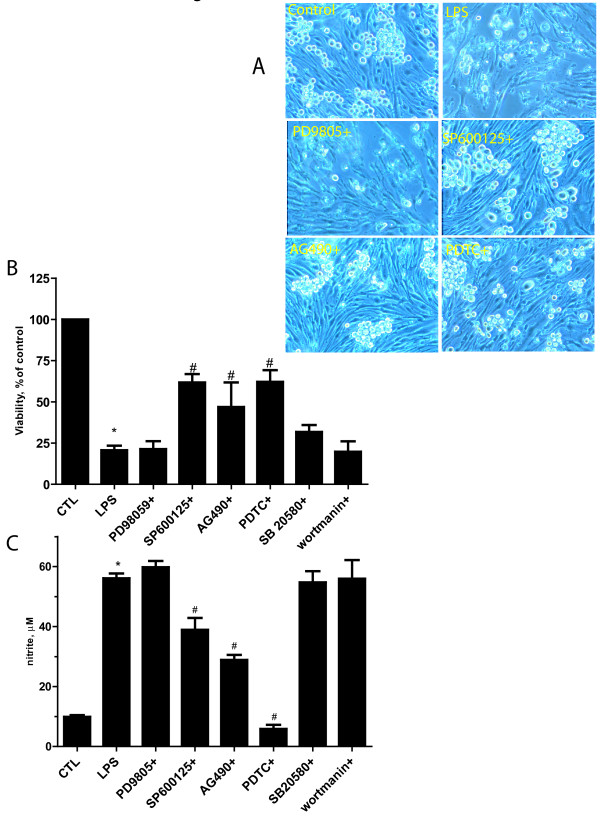
**NF-κB, JAK-STAT and JNK kinase inhibition prevent LPS- induced iNOS and protect from LPS -induced injury in BV2 and bEND.3 coculture model**. Panel A: LPS treatment of bEND.3/BV2 cocultures (LPS) increased cell death and disruption of bEND.3 monolayers compared to control cocultures (Control). Treatment with inhibitors to block JNK (SP600125), JAK-STAT (AG490) or NF-κB (PDTC) reduced this disruption, whereas treatment with a MEK1 inhibitor (PD98059) did not. Panels B & C summarize the effect of these various inhibitors on LPS-induced cell viability (B) and NO accumulation (C). These data show that inhibition of JNK, JAK-STAT and NF-κB improve cell viability while decreasing NO accumulation, whereas inhibition of MEK1, p38 MAPK (SB 203580) and PI3K (wortmannin) do not. CTL: control cultures treated with vehicle. (n = 12 *independent observations*), *P < 0.05vs. control, # P < 0.05 versus LPS).

We further explore the signaling pathways involved in NO activation in BV2 cells, and that this correlates to bEND.3 cell death in our coculture model (Figure 8). JNK, JAK-STAT and NF-κB inhibition in cocultures protected cells from LPS while reducing NO accumulation. The extent of NO accumulation in cocultures mirrored that seen in BV2 cells alone, with the most robust effects observed by inhibition of NF-κB and JAK-STAT, but some effect was also observed by JNK inhibition as well. There was no effect on cell death using inhibitors of MEK1, PI3K or p38 MAPK.

## Discussion

We previously showed that microglia increase injury to BBB components following experimental stroke and ischemia-like insults [6]. We now show that microglial activation by LPS induces injury to endothelial cells, and this LPS effect requires the presence of microglia. The mechanism of this effect appears to be mediated through NF-κB, JAK-STAT and JNK, rather than ERK, p38 MAPK or PI3K. The lack of effect through p38 MAPK is somewhat surprising given prior work emphasizing the importance of this pathway in inflammatory signalling [20,21]. Reasons for this discrepancy are unclear, but could be due to the model system studied. Regardless, these observations have therapeutic implications for a variety of conditions where immune cell injury to brain endothelial cells contributes to brain pathology. Since endothelial cell tight junctions make up the basis of the BBB, damage to these cells would lead to leakage of brain vessels permitting seepage of potentially toxic serum proteins and blood cells into the brain tissue. Blood elements are known to exacerbate injury through vasogenic edema and direct tissue damage [22].

TLR4, the receptor to which LPS binds has been shown to participate in a variety of central nervous system insults not necessarily related to infection [23]. Mice deficient in TLR4 have better outcomes following experimental stroke and decreased inflammatory responses [24-29], and the presence of TLR-4 on monocytes in stroke patients correlated to the extent of ischemic brain injury [30]. This would suggest that TLR4 signaling plays a significant and detrimental role in brain ischemia. While its precise ligand has not yet been identified in non-infectious conditions, a few studies have implicated heat shock proteins (HSPs), which may bind TLR4 [31], although these observations could be explained by contamination of HSP preparations by LPS or other proteins [32,33]. Regardless, TLR4 signalling is now known to contribute to a variety of non-infectious brain pathologies.

These studies build on our prior observations that microglia activated by ischemic stimuli are toxic to constituents of the blood brain barrier [6]. Here we used microglial BV2 cells stimulated with LPS, as an agonist model of TLR4 activation. We found that *LPS *stimulation of microglia was toxic to endothelial cells, suggesting one pathway that might explain the toxicity observed in our ischemia model. As expected, LPS could only stimulate microglia, but not endothelial cells. LPS also directly induced cell death in microglia, but not endothelial cells. However, LPS could only injure endothelial cells when cocultured with microglia which is not entirely surprising since endothelial cells are not known to express TLR4 receptors. Nevertheless, this observation underscores the toxic potential of microglia on these cells. The amount of cell death in the endothelial cell-microglial cocultures was mostly due to endothelial cells based on morphological and immunohistochemical evidence provided here. Microglia suffered a relatively low level of cell death, compared to endothelial cells. Further, *the endothelial monolayer integrity was markedly disrupted*. Thus, LPS induced factors in the BV2 cells which are cytotoxic. *Our data also suggest that as NO generation is suppressed, BV2 viability increased in parallel in most cases. The exceptions were indomethacin which did not suppress NO but did improve BV2 cell viability, minocycline which reduced both BV2 cell viability and NO generation, and NOHA which had no effect on either NO or viability*.

These data agree with prior studies showing that cytokine activated microglia are toxic to neurons and oligodendrocytes [34,35]. The toxic factors elaborated by activated microglia appear to include reactive nitrogen (RNS) and oxygen species (ROS), as pretreatment with NOS inhibitors (L-NMMA and aminoguanidine) and ROS inhibitors (apocynin and allopurinol) markedly reduced endothelial disruption in this *in vitro *model. Since we also found that SIN-1 was highly effective in inducing dose dependent NO accumulation and death, much like that seen with LPS, we suggest that microglial generation of RNS and ROS may further lead to the generation of peroxynitrite, another highly reactive compound.

**Figure 9:**
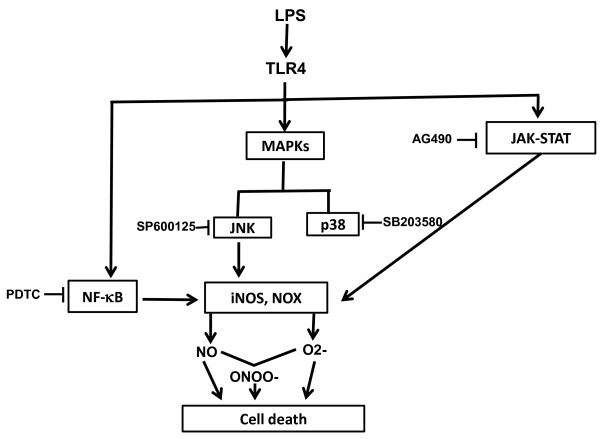
**Proposed mechanism of LPS signaling in microglia leading to endothelial, and microglial cell death:**. *LPS binds toll-like receptor 4 (TLR4) on the surface of microglia leading to signaling in several pathways., NF-κB, the MAPKs and JAK-STAT. MAPKs then activate JNK (JNK kinase) and the p38 MAPK (p38). NF-κB, JAK-STAT and to a lesser extent, JNK lead to upregulation of immune factors iNOS and NADPH oxidase (NOX). These factors lead to the production of nitric oxide (NO) and superoxide (O2-), respectively. These molecules are themselves known to be directly cytoxic, but may also combine to form peroxynitrite (ONOO-) which can also kill cells. Also indicated in the figure are the NF-κB, p38 MAPK, JAK-STAT and JNK inhibitors studied. (PDTC = pyrrolidinecarbodithoic acid)*.

To further explore the mechanisms of LPS mediated injury in our model, we studied several different signal transduction pathways known to be activated by TLR4 signalling through LPS. Interestingly, we found that several downstream kinase and transcription factors (JNK, p38 MAPK, JAK-STAT and NF-κB) were activated. *These factors could then lead to upregulation of immune molecules including iNOS and NADPH oxidase (NOX) which then generate NO and superoxide, respectively. These factors singly, as well as peroxynitrite, generated from NO and superoxide, are known to be cytotoxic (Figure *9). Interestingly, activated p38 MAPK did not appear to participate in cell survival or NO generation. LPS induced marked nuclear translocation of NF-κB in microglia and its inhibition by PDTC suppressed NO generation, but did not improve BV2 cell viability. *Our data indicate that while multiple transcription factor pathways are upregulated by LPS, NF-κB and JAK-STAT appear to be the ones involved in NO generation in BV2 cells, as well as JNK to a lesser extent. The differential effects of NF-κB versus JAK-STAT and JNK inhibition on cytoprotection also indicate that inhibition of microglial activation does not always correlate to their viability*.

However, when cultured with endothelial cells, NF-κB inhibition improved overall coculture viability and decreased NO. Thus, NF-κB may be essential for microglial viability while also suppressing its activation. Since microglia are essential to other aspects of tissue viability such as protecting against microbial invasion and assist in recovery and repair [36,37], a therapeutic intervention that suppresses microglial cytotoxicity while preventing microglial death may be more desirable.

JAK-STAT signaling promotes and modulates inflammatory processes. Phosphorylated JAKs lead to the activation of several substrates and provides docking sites for STATs, which in turn become phosphorylated for full STAT activity. Phosphorylated STATs are released from the receptor complex and form dimers which translocate to the nucleus. Once in the nucleus, they directly bind to the promoter region of specific target genes, many of which are involved in immune responses [38,39]. When we inhibited JAK-STAT in our model, not only did we observe decreased NO generation, but we also observed improved microglial viability. JAK-STAT inhibition also improved overall viability in the cocultures. Thus, JAK-STAT may be a preferred therapeutic target, as its inhibition appears to inhibit immune responses but does not destroy microglia while doing so.

MAPKs are important mediators involved in a variety of cell signalling functions, including inflammation [40]. The MAPK family includes p38, ERK and JNK, of which p38 and JNK are activated in response to environmental stress, whereas ERK is involved in growth responses. However, we did not observe any significant effect in our model by inhibiting these pathways, although there was a partial effect when blocking JNK. PI3K inhibition did not affect NO accumulation or cell death in our models, suggesting that it may not be an important downstream TLR4 target in cytoprotection.

We show that LPS activated microglia are toxic to endothelial cells, and in particular, targeting the JAK-STAT pathway in microglia would confer protection of both endothelial cells and microglia, and prevent microglial activation. This may be in preference to targeting NF-κB which appears to be toxic to microglia, and JNK, where *protection *was less robust. Thus, JAK-STAT inhibition *to prevent microglial toxicity *would have implications for preserving the BBB in relevant disease states such as sepsis and even non-infectious brain pathologies such as ischemia and trauma.

## Conclusions

LPS activated microglia are toxic to endothelial cells, and the pathways mediating this effect appear to involve NF-κB, JAK-STAT and JNK, rather than ERK, p38 MAPK or PI3K. Targeting the former pathways in microglia, especially JAK-STAT may be useful in preventing BBB disruption.

## Competing interests

The authors declare that they have no competing interests.

## Authors' contributions

RK carried out the cell culture, biochemical and immunoassays, the study design, the data analysis and drafted the manuscript. MY and RG help conceptualize the study, participated in its design and coordination, interpreted the data and critically shaped the manuscript draft. All authors read and approved the final version of the manuscript.
